# On the evolving open peer review culture for chemical information science

**DOI:** 10.12688/f1000research.7460.1

**Published:** 2015-11-25

**Authors:** W. Patrick Walters, Jürgen Bajorath

**Affiliations:** 1 Vertex Pharmaceuticals Incorporated, Boston, MA, USA; 2 Department of Life Science Informatics, B-IT, LIMES Program Unit Chemical Biology and Medicinal Chemistry, Rheinische Friedrich-Wilhelms-Universität, Bonn, Germany

**Keywords:** chemical information science, open peer reviews, review criteria, publication time lines, controversial views

## Abstract

Compared to the traditional anonymous peer review process, open post-publication peer review provides additional opportunities -and challenges- for reviewers to judge scientific studies. In this editorial, we comment on the open peer review culture and provide some guidance for reviewers of manuscripts submitted to the *Chemical Information Science *channel of *F1000Research*.

## Introduction

The *Chemical Information Science (CIS)* channel of *F1000Research* has been introduced as a publication platform ^1^ that covers all aspects of chemical information science ^2^ and positions the full spectrum of chemoinformatics approaches ^3^ within this broader context. The *CIS* channel ** specifically aims to attract high-quality manuscripts. Therefore, submissions to the *CIS* channel ** undergo a two-layer expert review, as described below. This editorial is intended to provide specific guidance for reviewers of studies published in the *CIS* channel.

## Pre-review and review

The review of papers submitted to the *CIS* channel ** takes place in two stages, an initial pre-review by members of the channel Editorial Board, followed by open peer review. Once a submission has been processed by *F1000Research* editorial staff and passed on to the guest editors, members of the channel Editorial Board evaluate a manuscript on the basis of its scientific potential to advance the field. This initial assessment (pre-review) is not meant to result in formal reviews, but a collection of expert opinions. The conclusions of the channel Editorial Board are then forwarded to the authors. If a positive pre-review consensus is reached or if views of the channel Editorial Board on a submission remain controversial, the paper is published in the *CIS* channel and reaches the stage of open peer review. If a negative pre-review consensus is reached by the channel Editorial Board, the manuscript is not published in the *CIS* channel (but the authors have still the opportunity to publish their work in *F1000Research*).

Upon publication of a paper in the *CIS* channel ** the authors are asked to make reviewer suggestions; members of the channel Editorial Board may suggest additional reviewers. Authors must agree with the final reviewer line-up before *F1000Research* editorial staff initiates the post-publication review. The review, approval, and indexing process of *CIS* channel publications follows standard *F1000Research* procedures.

## Open peer review specifics

The open post-publication peer review presents referees with different opportunities and challenges compared to the conventional anonymous peer review process. *The general philosophy of open peer review is that the reviewer identity will be disclosed and the review directly presented to the scientific community including the authors (without editorial interference). In addition, authors and readers have the opportunity to comment on reviews.* In the following, we provide some specific comments and guidelines for reviewers of *CIS* channel publications.

## Guidelines

(1) The primary function of a review is to evaluate whether a given study is *scientifically sound*, *understandably presented*, and *reproducible*. Frequent lack of reproducibility is a major issue concerning chemoinformatics publications in many journals ^4^. Reviewers of *CIS* channel publications must determine whether data and methods used in a given study are accessible to the scientific community and that sufficient details are provided to reproduce reported calculations and re-implement a method (provided an implementation of the method is not made available as part of the study). Answering these key questions should directly lead the reviewer to conclude that a study should be “approved”, “approved with reservations”, or “not approved”. *The reviews can be brief as long as they clearly address the key questions*.

(2) Because these questions are among those already considered during the pre-review, *members of the channel Editorial Board are encouraged to convert/extend pre-review comments into a post-publication review*. This will inevitably reduce the time required for a *CIS* channel publication to reach approval status (or a status requiring revisions).

(3) Open peer reviews not only provide feedback for authors, they also help to position a paper within the *CIS* channel and spark the interest of the scientific community. As such, these reviews and subsequent on-line discussions become an essential part of a publication. *An open review process can also dramatically reduce the time between submission, publication, and indexing of a paper, thus supporting its dissemination.* Short review times are highly desired and particularly important for *Data* or *Method* articles, which often report tools made freely available to the scientific community. In addition, short review times are an additional attraction for authors to submit their work to the *CIS* channel.

(4) Of course, reviewers are at liberty -and encouraged- to *provide detailed reviews, which might also suggest more or less extensive revisions*. This particularly applies to *Research Articles* or *Reviews*. It is also appropriate to further extend reviews of a paper after approval status is reached. This can be accomplished, for example, by *adding comments to initial reviews*. We expect that diligent authors will take reviewer comments seriously and submit revisions and/or responses. If authors disagree with review conclusions or requests, they can comment on them and articulate their viewpoints. Authors are specifically encouraged to publish appropriate revisions in a second version of their manuscript. If they do or do not take reviews seriously will be clearly visible to the scientific community; another bonus of an open review culture.

(5) *Answering the key questions if a study is scientifically sound, clearly presented, and reproducible in a timely manner becomes especially important for off-the-beaten path contributions*, which are explicitly encouraged by the *CIS* channel. For example, such papers might introduce novel, provocative, and/or controversial concepts that are far from being established, report negative results, or principal shortcomings of current methods. In such cases, views of authors, reviewers, and readers might often differ. Regardless, conceptually novel or controversial investigations that are viewed differently must still be scientifically sound. Even in the presence of different opinions, a careful assessment of the key questions is an essential task for reviewers of such *CIS* channel publications.

## Conclusions

We hope that our comments will help to foster a culture of open peer review, for the *CIS* channel and beyond. As discussed, open peer reviews are not written for editors but directly address authors and the scientific community. As such, they become a part of a publication and are thought to make important contributions to the further scientific development of our field. Open peer reviews must evaluate the key questions whether a publication is *scientifically sound*, *understandably presented*, and *reproducible* and may go well beyond answering these questions. Short review times are important when a paper is presented to the scientific community. Timely reviews, be they positive or negative, indicate that studies are taken seriously, make publications more interesting to readers, and help to disseminate them. Open peer review catalyzes scientific perception. The scientific community has the last word.
